# Toxocariasis in children: seroprevalence after 15 years in a major city in Brazil

**DOI:** 10.3389/fped.2025.1663016

**Published:** 2025-11-19

**Authors:** Viviane dos Santos Vaccaro Lima, Isabella Braghin Ferreira, Gabriela Geraldi da Silva Rapchan, Susana Zevallos Lescano, Gabriela Rodrigues e Fonseca, Rogerio Giuffrida, Louise Bach Kmetiuk, Alexander Welker Biondo, Vamilton Alvares Santarém

**Affiliations:** 1 Graduate College in Animal Science, University of Western São Paulo (Unoeste), Presidente Prudente, São Paulo, Brazil; 2 Graduate College in Health Sciences, University of Western São Paulo (Unoeste), Presidente Prudente, São Paulo, Brazil; 3 Laboratory of Medical Investigation, Clinical Hospital of the University of São Paulo, FMUSP, São Paulo, Brazil; 4 Center of Environmental Health, Municipal Secretary of Health, Curitiba, Paraná, Brazil; 5 Graduate College of Cell and Molecular Biology, Department of Veterinary Medicine, Federal University of Paraná (UFPR), Curitiba, Paraná, Brazil

**Keywords:** epidemiology, parasite, pediatric infection, toxocariasis, zoonosis

## Abstract

**Introduction:**

Children have a potentially greater exposure to *Toxocara* spp. infection via egg ingestion due to playground soil contact. Understanding the associated risk factors may help to prevent infection in children; however, there were no studies that monitored urban seropositivity over time. Therefore, we aimed to assess *Toxocara* seroprevalence and the associated risk factors in children who regularly accessed the Public Health System in a major city area in southeastern Brazil over a period of 15 years (2008–2023).

**Methods:**

A cross-sectional study was designed in 2023–2024 to screen for anti-*Toxocara* antibodies in children through an enzyme-linked immunosorbent assay alongside an epidemiological questionnaire to assess health status and living conditions. Univariate and multivariate logistic regression analyses were used to investigate the factors associated with seropositivity in children.

**Results:**

In total, 30/260 [11.5%; 95% confidence interval (CI): 8.2%–16.0%] children were found to be seropositive for *Toxocara* spp. Boys were at a higher exposure risk [odds ratio (OR): 4.4] to toxocariasis than girls. Having a mother with a graduate degree (OR: 0.21; *p* = 0.016), receiving drinkable water supply (OR: 0.29; *p* = 0.017), and having hygienic habits of washing hands before meals (OR: 0.32; *p* = 0.033) were protective factors. The findings presented no significant difference (*p* = 0.879) when compared to those of a previous 2008 serosurvey, with 28/252 (11.1%; 95% CI: 7.8%–15.6%) seropositive children.

**Discussion:**

*Toxocara* seroprevalence in children living in an urban area did not significantly change over the 15 years since the previous serosurvey was conducted. As no mitigatory actions were carried out between the surveys, we speculate that environmental contamination and child exposure to *Toxocara* spp. remained steady. However, future surveys should include soil detection of *Toxocara* spp. to fully establish the role of environmental contamination over time. In conclusion, although toxocariasis in children may not increase over time, such seroprevalence may not decrease either, persisting (even in low levels) for long periods.

## Introduction

Human toxocariasis is a zoonotic helminth infection primarily impacting lower-socioeconomic populations living in tropical and subtropical countries (1). Toxocariasis has been classified as a neglected parasitic disease and targeted for public health action by the Centers for Disease Control and Prevention (2), alongside Chagas disease, cyclosporiasis, cysticercosis, toxoplasmosis, and trichomoniasis. The health burden of common toxocariasis in humans has been estimated to be 23,084 disability-adjusted life years lost annually in 28 selected countries (3). Based on an average global seroprevalence rate of 19%, an estimated 91,714 disability-adjusted life years are lost annually due to toxocariasis across affected countries; of these, 40,912 are related to less severe forms, i.e., common toxocariasis, and 50,731 to cognitive impairment in children. The estimated economic impact in Brazil, adjusted for purchasing power parity, was $283,389,884 over a 10-year period, a burden equivalent to that reported in Nigeria, India, the UK, Romania, and Ireland (3).

Human toxocariasis is mainly acquired through the ingestion of *Toxocara canis* and *Toxocara cati* eggs that have been shed into contaminated soil via the feces of dogs and cats, which act as definitive hosts (4, 5). *Toxocara* spp. infection may lead to a self-limiting febrile illness (common toxocariasis) in some patients or an asymptomatic (covert toxocariasis) infection. In addition, human toxocariasis may remain subclinical or lead to mild, moderate, or severe symptoms, including larva migrans syndromes (visceral and ocular) and neurotoxocariasis (4, 6).

Despite mostly affecting vulnerable populations in underdeveloped and developing countries, human toxocariasis remains a devastating zoonotic infection in children living in the USA (7). The disease is associated with urticaria (8), bronchial asthma and pneumonia (9), ocular impairment (10), and cognitive function decline (11) in children. Children are considered to be more exposed to infection through the ingestion of soil contaminated with *Toxocara* spp. eggs (4), along with direct contact with dogs and cats (12), particularly puppies and kittens (13).

According to a recent meta-analytic study, the estimated global seroprevalence of anti-*Toxocara* spp. antibodies among children is 25.0% [95% confidence interval (CI) 22%–29%], particularly in boys (14). Risk factors for toxocariasis have been reported worldwide, including caregiver education, contact with dogs/cats, and improper handling of pet feces associated to seropositivity in Vietnam (15) and drinking unpurified water in Iran (16). Higher risk was reported in asthmatic children with a recorded history of contact with soil and pets as compared to control and pneumonic groups in Egypt (9). In Brazil, associated risk factors for toxocariasis include family income (up to one minimum wage), the presence of a dog, the habit of playing with soil/sand, and eosinophilia (17).

Although the prevalence of toxocariasis in Brazil has been estimated in meta-analytic studies to be 28.0% (95% CI: 22–34) in the general population (18) and 39.3% (95% CI: 29.8–48.7) in children (14), a higher seroprevalence of 63.6% was reported in infants in socioeconomically vulnerable conditions in northeastern Brazil (19). In a previous study conducted in southeastern Brazil in 2008, 28/252 (11.1%) children were seropositive for *Toxocara* spp. seropositivity was observed in 12/126 (9.5%) children from a middle-income background and in 16/126 (12.7%) children from a lower-income background, indicating an inversely proportional connection to family income (20). In addition, higher household income and female gender were significant protective factors for both subgroups (20). Although the knowledge of toxocariasis occurrence and associated risk factors is important for understanding the disease mechanisms of transmission, to our knowledge, no study has been conducted on the dynamics of toxocariasis in children over time. Therefore, in this study, we aimed to assess the seroprevalence and associated risk factors for toxocariasis in children who visited the Public Health System between 2008 and 2023 in the same area of southeastern Brazil that was studied previously.

## Methods

### Ethics statement

This study was approved by the Ethics Committee of the University of Western São Paulo (protocol 8,294), and the protocol has been submitted to the National Commission of Ethics in Research (CAAE: protocol 73476123.2.0000.5515). For data collection, each guardian was informed about the confidentiality of participant identity and the right to refuse participation at any time. The guardian formalized the authorization by signing the Free and Informed Consent Terms form in compliance with Resolution No. 441/2012 of the National Brazilian Health Council. The anonymity of the participants was ensured by assigning a random number that was used to identify each sample and metadata, according to criteria adopted elsewhere (21). Information produced by the researchers regarding toxocariasis was provided to each child enrolled in the study (Supplementary Figure S1).

### Study design

**Figure 1:**
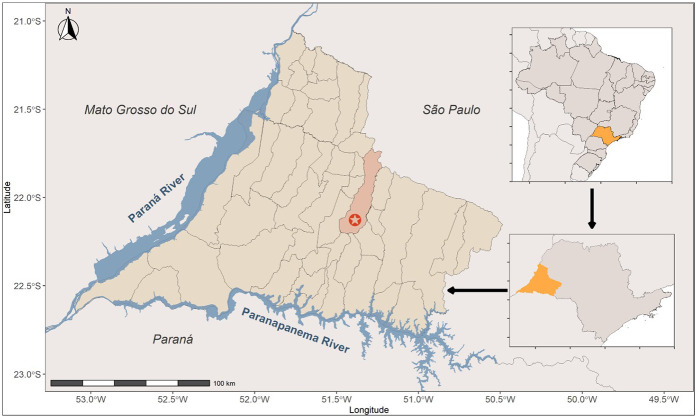
Map of Brazil showing the study location. The municipality of Presidente Prudente is presented, with the city indicated by a red star. The state of São Paulo (upper right) and its western region (bottom right) are highlighted in yellow.

This cross-sectional study was designed to detect anti-*Toxocara* spp. antibodies and gather information from a pediatric population accessing the Brazilian Public Health System. The participants were recruited during routine examinations requested by the public health system at the Dr. João Carlos Grigoli Clinical Analysis Laboratory at the University of Western São Paulo between November 2023 and April 2024. The study was conducted in the municipality of Presidente Prudente (22°7′16.5540ʺS and 51°23′0.2400ʺW), São Paulo state, southeastern Brazil (Figure 1). Presidente Prudente is a major Brazilian city, currently ranked 25th (top 0.5%) in the city human development index (HDI) (0.806, very high), 138th (top 2.5%) in population with 225,000 inhabitants, and 159th (top 3.0%) in gross domestic product out of 5,565 municipalities in Brazil.

The study was conducted at a public prenatal referral health establishment located in the municipality of Presidente Prudente (22°7′16.5540ʺS and 51°23′0.2400ʺW), São Paulo state, southeastern Brazil.

### Sample size calculation

Using the Scalex SP calculator (22), we calculated the sample size of 259, adjusted to 260 assuming the upper 95% confidence limit (19.6%) of the prevalence in children in Presidente Prudente as observed in a previous study (20). We assumed a margin of error of ±5% in estimating the prevalence with 95% confidence and a 5% potential loss.

### Inclusion and exclusion criteria

Children aged 1–11 years, regardless of sex, whose legal guardian (>18 years) provided written consent were included in the study. All children were randomly invited to participate during routine visits to the Brazilian Public Health System. The age of the children included was established based on the Brazilian Statute of the Child and Adolescent (Law number 8,069, 13 July 1990) (23), which considers someone to be a child if they are ≤12 years of age. Children living outside Presidente Prudente and/or children who did not accept the invitation to participate in the study were excluded.

### Sociodemographic data

To gather information on socioeconomic status, residence, contact with pets (dog or cats), and hygienic habits and practices (Table 1), a semistructured questionnaire was completed by the guardian. The data were gathered in a spreadsheet (Excel) to evaluate the associated risk factors for toxocariasis.

**Table 1:** Data for assessing the potential children's exposure to toxocariasis.

Topics	Gathered information
Socioeconomic and demographic characteristics	Sex, age, mother's educational level, monthly household income, household location
Residence	Drinkable water, unpaved yard
Domestic animals	Owning dog, dog contact, owning cat, cat contact
Hygienic habits and practices	Washing hands before meals, contact with soil, playing on public parks, ingestion of raw meat, biting nails (onychophagy), and pica behavior

### Collection of blood samples

Blood samples (3.0 mL) were collected by an authorized health professional belonging to the health establishment staff once per child via peripheral venipuncture using commercial vacuum tubes (Vacutainer, BD Co., Curitiba, Brazil). Blood samples were then centrifuged at 1,295 *g* for 5 min; serum was collected and frozen (−20°C) until the test.

### Screening anti-*Toxocara* immunoglobulin (Ig)G antibodies

#### *Toxocara* antigen for enzyme-linked immunosorbent assay test

The antigen used for detecting anti-*Toxocara* IgG antibodies was obtained from female *T. canis* adult specimens spontaneously shed by naturally infected puppies. The roundworms were rinsed in 1% sodium hypochlorite (5 min) and the washed with 0.9% saline (3 min). After removing debris, the anterior third of the female worm was dissected to obtain eggs. Protocols previously described were employed to produce *T. canis* excretory-secretory (TES) antigen *in vitro* (24) and concentrate the protein (25).

Owing to the possibility of cross-reactivity with other ascarids, serum samples were preincubated (30 min at 37°C) with *Ascaris suum* adult worm extract solution (25.0 μg/μL) in 0.01 M phosphate-buffered saline (PBS, pH 7.2) containing 0.05% Tween 20 (PBS-T) (Sigma, St. Louis, MO, USA), following a published protocol (24).

#### Serological testing

IgG antibodies against TES were detected through indirect enzyme-linked immunosorbent assay (ELISA) at a dilution of 1:200. Polystyrene 96-well microtiter plates (Corning, Costar, NY, USA) were coated (1 h at 37°C) and then incubated for 18 h at 4°C, with 2.0 μg/µL per 100 µL/well of TES antigens in 0.06 M carbonate-bicarbonate buffer (22, 23), at pH 9.6. Then, they were blocked (1 h at 37°C) using 3% Molico® skimmed milk 5% PBS-T. After blocking, the wells were washed three times with PBS-T (5 min each). Subsequently, 100 µL of the sera preadsorbed with *A. suum* somatic antigen was added to the wells in duplicate. The plates were incubated for 1 h at 37°C, washed three times with PBS-T (5 min each), and antihuman IgG (Fc-specific) peroxidase antibody—produced in goat (Sigma A6029)—was added at a 1:10,000 dilution (45 min at 37°C). The plates were then washed three times (5 min each).

The reaction was initiated using the substrate o-phenylenediamine (0.4 mg/mL, Sigma), and it was stopped by adding 2 N sulfuric acid. Absorbance was read at 492 nm. A serum previously shown to be non-reactive (negative control) and a known reactive serum (positive control) were tested in each plate. Antibody levels are expressed as reactivity indexes, which were calculated as the ratio between the absorbance values of each sample, and the cutoff value was 0.285.

### Data analysis

All statistical analyses were performed using R v.4.1.1 (R Core Team, 2022). To assess risk factors related to seropositivity, the outcome data were categorized (variables shown in Table 1). Then, univariate analysis (Pearson chi-square or Fisher's exact test) was performed. Variables presenting a significance of <0.20 in the univariate analysis were considered suitable for logistic regression analysis (multivariate analyses) to assess the risk/protective factors associated with the likelihood of *Toxocara* spp. seropositivity. To improve the final model, the predictor variables were tested for collinearity and the presence of influential values. Variables with a high degree of collinearity were excluded from the logistic model (variance inflation factor >4.0). From the regression coefficients for each predictor variable, odds ratios (ORs) were estimated per point with 95% CIs. The best-fitting model was considered the one that included significantly associated variables (*p*-value < 0.05) and minimized the Akaike information criterion.

A chi-square test for equality of proportions without Yates' continuity correction was performed to compare the seroprevalence for anti-*Toxocara* spp. antibodies at two distinct time points (2008 vs. 2023) within the target population. A 5% significance level was adopted for all statistical tests.

## Results

### Demographic characteristics of participants

In total, 260 individuals were sampled including 137/260 (52.7%) boys and 123/260 (47.3%) girls, aged 1–11 years (median: 6 years). In terms of the monthly family wage, 246 individuals responded to the questionnaire, while 14 individuals did not provide the information and were not included in familial income statistics. Thus, 112/246 (45.5%) guardians declared an income of less than one minimum wage (USD 312.00), 116 (47.2%) declared an income of up to USD 928.00, and 18 (7.3%) declared an income of more than USD 1,000.00. All participants had an indoor bathroom, and the majority (235/260; 90.4%) had a domestic sewage system. Approximately 50% (122/260; 46.7%) lived in a residence with a dirty yard.

### Seropositivity and risk factors for toxocariasis

**Figure 2:**
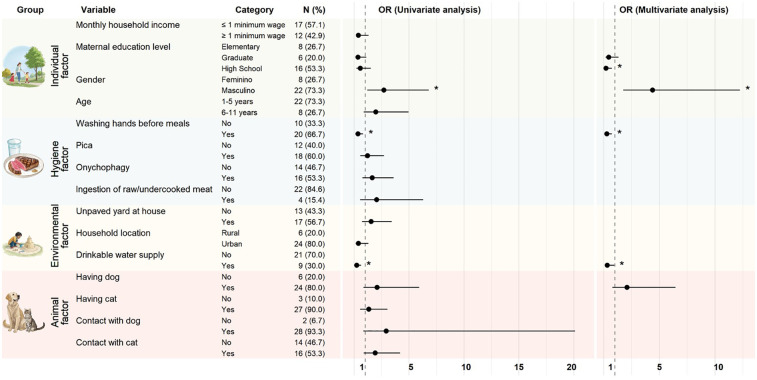
Risk/protective factors for *Toxocara* spp. seropositivity in a pediatric population (*n* = 260) assisted by the Public Health System, in southeastern Brazil, assessed using univariate and multivariate analyses. The vertical line with a value of 1 represents the line of null effect. OR is significant (*) if the mean with 95% confidence intervals (95% CI) does not touch the vertical line (value of 1). Risk factor: male sex (OR: 4.4; 95% CI: 1.8–12.2; *p* = 0.003). Protective factors: maternal education level (OR: 0.2; 95% CI: 0.1–0.7; *p* = 0.016), washing hands before meals (OR: 0.3; 95% CI: 0.1–0.7; *p* = 0.008), and drinkable water supply (OR: 0.3; 95% CI: 0.1–0.9; *p* = 0.033).

Anti-*Toxocara* spp. antibodies were detected in 30/260 children (11.5%; CI 95%: 8.2–16.0%). The final logistic regression model revealed that boys were 4.4-fold more likely to be seropositive than girls. Having a mother with a graduate degree (OR: 0.2; *p* = 0.016), access to a drinkable water supply (OR: 0.3; *p* = 0.033), and washing hands before meals (OR: 0.3; *p* = 0.008) were protective factors for toxocariasis (Figure 2).

The AI service Recraft V3 (26) text-to-image model (October 2024) was used to produce the four illustrations in Figure 2. The specific prompts used for generating the images were as follows: Figure 2, column 1, row 1—“Mother and children in a park, vector, realistic, white background”; Figure 2, column 1, row 2—“Beef on a plate with a cup of water, vector, realistic”; Figure 2, column 1, row 3—“A child playing in a park with a sandcastle, realistic drawing, white background”; and Figure 2, column 1, row 4—“Dog and cat, realistic vector, white background.”

Although the univariate analysis included having dog in the logistic regression, this variable was not considered significant in the final model (*p* = 0.162). Contact with a dog or a cat was considered to be suitable for inclusion in the multivariate analysis but was not retained in the final logistic regression model. The other evaluated variables were not significant in the univariate analysis (Supplementary Table S1): monthly income (*p* = 0.267), living in a house with an unpaved yard (*p* = 0.346), playing on public playground (*p* = 0.893), having a cat (*p* = 0.654), onychophagy (*p* = 0.27), pica (*p* = 0.764), raw/undercooked meat consumption (*p* = 0.268), and cat ownership (*p* = 0.654). The performance of the final regression model was considered to have a good to very good discriminative power (AUC: 79.8%; 95% CI: 71.2–88.4) (Supplementary Figure S2).

## Discussion

The present study reassessed the seroprevalence and risk factors of toxocariasis in a pediatric population in southeastern Brazil who accessed services through the Public Health System. The seropositivity and 95% confidence interval (28/252; 11.1%; 95% CI: 7.8–15.6) were highly similar to those observed previously in the same study area (20), suggesting the stability of *Toxocara* spp. seroprevalence. Furthermore, the male gender was considered to be a risk factor for *Toxocara* seropositivity in both the previous (20) and present studies.

The seroprevalence of *Toxocara* spp. infection observed in the present study (11.5%; 95% CI: 8.2–16.0) was lower than the estimated seroprevalence among children worldwide (25%; 95% CI: 22–29) and in Brazil (39.3%; 95% CI: 29.8–48.7) (14). In Brazil, the seroprevalence ranges between 4.2% (7/167) in the south (27) and 63.6% (503/791) in the northeast (19). Our results contradict those of a meta-analysis which revealed a decrease in *Toxocara* spp. seroprevalence in children over time, estimated at 33.6% (23.3–44.0) in 2000–2005, 23.3% (15.5–31.1) in 2006–2011, 29.1% (22.0–36.2) in 2012–2017, and 10.6% (7.08–13.4) in 2018–2021 (14). One of the reasons for such a decrease—according to the latest authors—is the level of health knowledge of the population.

Some factors may influence the rates of seropositivity in the population, including a low HDI, income level, latitude, and high environmental temperature (5). Thus, regional socioeconomic and climatic differences may explain the wide range of seropositivity in Brazil. In the present study, approximately 50% of the population lived with more than one monthly salary, but monthly household income was not associated with seropositivity. This result contrasts with the results of a serosurvey on schoolchildren in São Paulo, the largest city of Brazil (28), and the previous study conducted in Presidente Prudente (20), in which the frequency of *Toxocara* spp. infection was higher in children whose families had the lowest monthly income. In contrast with previous findings, household income in the present study was not associated with toxocariasis seropositivity. This outcome could be a consequence of the city's high HDI, which may have evenly impacted the overall population (5). Thus, the steady low toxocariasis seropositivity over time in the children observed in our study could be the result of adequate population hygiene habits and water supply.

Sanitation conditions have been linked to toxocariasis, with conflicting results in pediatric populations, with poor sanitation associated (29) or not (30) with seropositivity. In Argentina, highly unsanitary conditions and lack of water supply and sewer networks led to a high toxocariasis seroprevalence in abandoned, institutionalized children (31). In Brazil, adequate sanitary facilities were considered to be a protective factor, leading to the relatively low seroprevalence previously observed in children from Presidente Prudente (32). In addition, in the present study, access to drinkable water supply was revealed to be a protective factor for seropositivity, corroborating the results of a population-based study of children in Amazonia, northern Brazil, in which multivariate analysis revealed piped water inside the household to be a protective factor (33). Furthermore, in Iran (16) and Thailand (34), consumption of untreated water was considered a risk factor for toxocariasis in children.

Hygienic habits and residence conditions contribute to *Toxocara* spp. infection (35). The habit of washing hands before meals was a protective factor for toxocariasis in the present study. This finding is in line with those of similar serosurveys involving children in China (36) and Brazil (30). Although poor hygiene habits, such onychophagy and pica, may increase the risk of *Toxocara* spp. infection via egg ingestion (35, 37, 38), these habits were not associated with seropositivity in the present study. Poor hygienic habits are considered to be more common in socioeconomically disadvantaged children (30). A study conducted in Saudi Arabia to evaluate the level of handwashing knowledge, attitudes, and practices among school students showed a positive correlation between the education level of the mother and hand hygiene practices (39). Thus, the influence of maternal education on hygienic practices observed in the present study should be considered another reason for the seropositivity that we observed. In a previous study conducted in northeastern Brazil, low maternal education was associated with a higher seroprevalence to *Toxocara* spp. and reflected the family hygiene habits (40). Likewise, a higher educational level was previously reported to be a protective factor for toxocariasis among adults in vulnerable populations in Brazil, including people experiencing homelessness (41) and inmates (32).

Food-borne transmission may also play a role in human toxocariasis. There is a lower risk of consuming larvated eggs in unwashed spinach than in undercooked meat (42). Such research highlights the importance of reducing environmental *Toxocara* spp. contamination in farms and correctly cooking meat to prevent toxocariasis.

As observed both in this study and the previous study conducted in the same study area (20), boys were more likely to be seropositive for *Toxocara* spp. than girls. This finding corroborates the conclusions of a global meta-analysis on seroprevalence for toxocariasis in pediatric populations, which reported a higher prevalence in boys, potentially linked to social behaviors and higher exposure to *Toxocara* spp. eggs (14), previously related to pica, geophagia, and poor hygiene practices (43, 44). A higher risk of contact with the soil and easy contamination—due to the engagement of boys in outdoor activities and games—have been reported as contributing factors for infection by other pathogens (45). These authors observed significantly more *Escherichia coli* on the hands of boys than on the hands of girls because boys were less likely to wash their hands with soap after playing outside. Studies have shown a positive association between seropositivity and children playing in public squares (46), as well as living in homes with a dirty backyard (28). In the present study, neither of these variables (playing in public parks or the presence of a dirty backyard) was associated with seropositivity. A study conducted in Presidente Prudente verified a high number of contaminated public parks (24/25; 96.0%) and reported that the climatic conditions and soil characteristics in its parks were conducive to the year-round maintenance of *Toxocara* spp. eggs (47).

People living in urban areas are reportedly more likely to be seropositive due to higher exposure to toxocariasis agents (16, 48). However, in the present study, no differences were noted when comparing children living in urban and rural locations, corroborating the findings of a nationwide serosurvey in Israel (49). According to these authors, the contamination of soil with *Toxocara* spp. eggs might be greater in urban areas and domiciled dogs, instead of stray dogs, may contribute as the primary source of human infection. In the present study, most of the studied population lived in urban areas; however, it is difficult to infer a reason justifying our findings.

Contact with cats or dogs was not associated with seropositivity for *Toxocara* spp. antibodies in children in this study, contrasting with findings from a meta-analysis of a moderate positive correlation between dogs and human prevalence and a significant positive correlation between cats and human prevalence (3). In addition, contact with dogs and cats was significantly associated with seropositivity, particularly in younger individuals and in regions such as the Americas, the Middle East, and the Western Pacific, according to another meta-analysis (12). A previous study conducted at the Veterinary Teaching Hospital in Presidente Prudente reported that only 11/165 (6.7%) dogs were contaminated with non-embryonated *T. canis* eggs, suggesting low risk of toxocariasis transmission by direct contact in well-maintained dogs (50).

In this study, no information regarding the hygienic or health status of dogs or cats was collected. Nevertheless, independent of the significance of the presence of *Toxocara* spp. eggs on the hair, regular deworming and hygiene of pets are important considerations for minimizing environmental contamination. As a general guideline, dogs and cats older than six months should be dewormed four times a year, particularly in endemic areas, to reduce the toxocariasis risk due to pet contact and environmental contamination (12, 51). Although pet neutering may not directly reflect on toxocariasis transmission, a decrease in stray pet populations can indirectly reduce environmental contamination, primarily in public parks (52). In addition to pet neutering and deworming, general hygiene measures may decrease exposed populations to egg ingestion from contaminated environments, particularly children (53).

The ingestion of raw/undercooked meat from paratenic hosts has been reported to be responsible for toxocariasis transmission in Brazil, with sources including sheep (54, 55), chickens (56, 57), and cattle (58). However, no association with seropositivity was observed in the present study. Although the consumption of raw/undercooked meat in children could influence seropositivity, supporting data are lacking. Thus, future investigations should assess the consumption of raw/undercooked meat as an associated risk factor for toxocariasis in children.

Regarding age, the frequency of seropositivity was higher in older (6–11 years) rather than younger (1–5 years) children, but no association to seropositivity was revealed in the logistic regression analysis. It has been stated that the behavioral habits of younger individuals increase exposure to contaminated soil. However, anti-*Toxocara* seropositivity increases with the age of children age due to repeated infection, leading to the persistence of antibodies (59).

Despite our promising results, the study has some limitations. First of all, as a cross-sectional study involving pediatric populations spread out over a period of time and relying on epidemiological questionnaires, inaccuracy or falseness of responses by guardians could have partially biased the results and statistical analysis. Furthermore, the findings reflect associations rather than causative factors. Second, it was not possible to verify the association between *Toxocara* spp. infection and different levels of exposure to pets, soil, and raw/undercooked meat. In addition, the ELISA assay (sensitivity 78.3%; specificity 92.3%) for antibody detection does not distinguish between recent and past toxocariasis infection due to IgG persistence. Although the absence of confirmatory tests, such as western blotting, may raise concerns about false positives, *A. suum* adsorption was used in previous studies conducted by our research group to prevent cross-reactivity (20, 32, 41, 56, 60). All children sampled were randomly invited to participate while attending routine examinations at the Brazilian Public Health Service. Unlike previous studies conducted by our research group in other vulnerable populations, this study did not include further access to pets of the participants for veterinary intervention, including deworming and vaccination. Furthermore, we could not assess environmental samples and contamination. Finally, no assessment of hematological results was possible, limiting the clinical interpretation of seropositive cases, as eosinophilia is an important laboratorial finding associated with toxocariasis in children (7, 13, 61). Future studies should include hematological markers (eosinophilia) and possibly clinical symptoms to strengthen the epidemiological interpretation, as well as environmental sampling (soil and playgrounds) to clarify the transmission routes.

In conclusion, the present study suggests that although toxocariasis in children may not increase over time, such seroprevalence may not decrease either, persisting (even in low levels) for long periods, particularly if no intervention has been made. Further studies should be conducted to fully establish the environmental stabilization of *Toxocara* spp. in such a scenario, when no mitigatory actions were taken over time. Future studies should also aim to screen toxocariasis in children in a One Health approach, with concomitant evaluation of household conditions, human and pet health status, dog/cat raising practices, and soil contamination.

## Data Availability

The original contributions presented in the study are included in the article/Supplementary Material; further inquiries can be directed to the corresponding author.

## References

[1] Winders WT Menkin-Smith L, editors. Toxocara Canis. In: StatPearls. Treasure Island (FL): StatPearls Publishing (2025). http://www.ncbi.nlm.nih.gov/books/NBK538524/ (Accessed June 14, 2025). 30860759

[2] CDC. Parasites. *Parasites* (2024). Available online at: https://www.cdc.gov/parasites/index.html (Accessed June 14, 2025).

[3] Antonopoulos A Giannelli A Morgan ER Charlier J. Quantifying the neglected: initial estimation of the global burden and economic impact of human toxocariasis. Curr Res Parasitol Vector-Borne Dis. (2024) 5:100180. 10.1016/j.crpvbd.2024.100180 38872976 PMC11169085

[4] Ma G Holland CV Wang T Hofmann A Fan C-K Maizels RM Human toxocariasis. Lancet Infect Dis. (2018) 18:e14–24. 10.1016/S1473-3099(17)30331-6 28781085

[5] Rostami A Ma G Wang T Koehler AV Hofmann A Chang BCH Human toxocariasis—a look at a neglected disease through an epidemiological “prism.” Infect Genet Evol. (2019) 74:104002. 10.1016/j.meegid.2019.104002 31412276

[6] Magnaval J-F Bouhsira E Fillaux J. Therapy and prevention for human toxocariasis. Microorganisms. (2022) 10:241. 10.3390/microorganisms10020241 35208697 PMC8875715

[7] Fortini MB Erickson TA Leining LM Robinson KM Carey MN Smith SJ Review of toxocariasis at a children’s hospital prompting need for public health interventions. Pediatr Infect Dis J. (2023) 42:862–6. 10.1097/INF.0000000000004042 37625080 PMC10754417

[8] Matos Fialho PM Correa CRS Lescano SZ. Seroprevalence of toxocariasis in children with urticaria: a population-based study. J Trop Pediatr. (2017) 63:352–7. 10.1093/tropej/fmw094 28077610

[9] Abd El Wahab WM Ali MI Ibrahim SS Mohamed YA Hamdy DA. Toxocariasis: potential association with bronchial asthma, and pneumonia among pediatric children. J Parasit Dis. (2023) 47:93–100. 10.1007/s12639-022-01543-w 36910311 PMC9998777

[10] Juárez XS Delgado M Matteucci ED Schiavino S Pasinovich ME García-Franco L Toxocariasis in children: analysis of 85 cases in a paediatric hospital in Argentina. Rev Chilena Infectol. (2021) 38:761–7. 10.4067/s0716-10182021000600761 35506849

[11] Gale SD Hedges DW. Neurocognitive and neuropsychiatric effects of toxocariasis. Adv Parasitol. (2020) 109:261–72. 10.1016/bs.apar.2020.01.009 32381201

[12] Merigueti YFFB Giuffrida R da Silva RC Kmetiuk LB Santos APD Biondo AW Dog and cat contact as risk factor for human toxocariasis: systematic review and meta-analysis. Front Public Health. (2022) 10:854468. 10.3389/fpubh.2022.854468 35836995 PMC9273826

[13] Bustamante J Sainz T Pérez S Rodríguez-Molino P Montero Vega D Mellado MJ Toxocariasis in migrant children: a 6 years’ experience in a reference pediatric unit in Spain. Travel Med Infect Dis. (2022) 47:102288. 10.1016/j.tmaid.2022.102288 35247580

[14] Owjinezhad D Abdoli A Rahmanian V Shaterian N Bahadory S Matin S Global seroprevalence of toxocara spp. in children: a systematic review and meta-analysis. Acta Parasitol. (2024) 69:164–74. 10.1007/s11686-023-00772-0 38195773

[15] Ha TV Vo TTN Dang DKH Tran YML Kim TV Le DH The seroprevalence of toxocariasis and related risk factors in children in Ho Chi Minh City, Vietnam: results from a school-based cross-sectional study. Trans R Soc Trop Med Hyg. (2024) 118:384–90. 10.1093/trstmh/trad102 38261661

[16] Foroutan M Soltani S Bahadoram S Maghsoudi F Kamyari N Haddadi S. Seroprevalence and risk factors of *Toxocara canis* infection in children aged 2–15 years from the southwest Iran. Comp Immunol Microbiol Infect Dis. (2022) 85:101801. 10.1016/j.cimid.2022.101801 35364396

[17] Cabral Monica T Evers F de Souza Lima Nino B Pinto-Ferreira F Breganó JW Ragassi Urbano M Socioeconomic factors associated with infection by *Toxoplasma gondii* and *Toxocara canis* in children. Transbound Emerg Dis. (2022) 69:1589–95. 10.1111/tbed.14129 33908184

[18] Ulloque-Badaracco JR Hernandez-Bustamante EA Alarcón-Braga EA Huayta-Cortez M Carballo-Tello XL Seminario-Amez RA Seroprevalence of human toxocariasis in Latin America and the Caribbean: a systematic review and meta-analysis. Front Public Health. (2023) 11:1181230. 10.3389/fpubh.2023.1181230 37441649 PMC10335805

[19] Silva MB Amor ALM Santos LN Galvão AA Oviedo Vera AV Silva ES Risk factors for Toxocara spp. seroprevalence and its association with atopy and asthma phenotypes in school-age children in a small town and semi-rural areas of Northeast Brazil. Acta Trop. (2017) 174:158–64. 10.1016/j.actatropica.2016.04.005 27080332

[20] Santarém VA Leli FNC Rubinsky-Elefant G Giuffrida R. Protective and risk factors for toxocariasis in children from two different social classes of Brazil. Rev Inst Med Trop S Paulo. (2011) 53:66–72. 10.1590/S0036-46652011000200002 21537752

[21] Bugeza JK Roesel K Mugizi DR Alinaitwe L Kivali V Kankya C Sero-prevalence and risk factors associated with occurrence of anti-Brucella antibodies among slaughterhouse workers in Uganda. PLoS Negl Trop Dis. (2024) 18:e0012046. 10.1371/journal.pntd.0012046 38498555 PMC10977895

[22] Naing L Nordin RB Abdul Rahman H Naing YT. Sample size calculation for prevalence studies using Scalex and ScalaR calculators. BMC Med Res Methodol. (2022) 22:209. 10.1186/s12874-022-01694-7 35907796 PMC9338613

[23] L8069. Available online at: https://www.planalto.gov.br/ccivil_03/leis/l8069.htm (Accessed June 14, 2025).

[24] Elefant GR Shimizu SH Sanchez MCA Jacob CMA Ferreira AW. A serological follow-up of toxocariasis patients after chemotherapy based on the detection of IgG, IgA, and IgE antibodies by enzyme-linked immunosorbent assay. J Clin Lab Anal. (2006) 20:164–72. 10.1002/jcla.20126 16874812 PMC6807646

[25] Lowry OH Rosebrough NJ Farr AL Randall RJ. Protein measurement with the Folin phenol reagent. J Biol Chem. (1951) 193:265–75. https://pubmed.ncbi.nlm.nih.gov/14907713/14907713

[26] Recraft|AI for designers, creatives, sellers, and teams. Available online at: https://recraft.framer.website/ (Accessed October 3, 2025).

[27] Guilherme EV Marchioro AA Araujo SM Falavigna DLM Adami C Falavigna-Guilherme G Toxocariasis in children attending a Public Health Service Pneumology Unit in Paraná State, Brazil. Rev Inst Med Trop Sao Paulo. (2013) 55:S0036. 10.1590/S0036-46652013000300009 23740017

[28] Alderete JM Jacob CM Pastorino AC Elefant GR Castro AP Fomin AB Prevalence of Toxocara infection in schoolchildren from the Butantã region, São Paulo, Brazil. Mem Inst Oswaldo Cruz. (2003) 98:593–7. 10.1590/S0074-02762003000500002 12973524

[29] Martínez M García H Figuera L González V Lamas F López K Seroprevalence and risk factors of toxocariasis in preschool children in Aragua state, Venezuela. Trans R Soc Trop Med Hyg. (2015) 109:579–88. 10.1093/trstmh/trv059 26217045

[30] Cassenote AJF Lima AR de A Pinto Neto JM Rubinsky-Elefant G. Seroprevalence and modifiable risk factors for Toxocara spp. in Brazilian schoolchildren. PLoS Negl Trop Dis. (2014) 8:e2830. 10.1371/journal.pntd.0002830 24874504 PMC4038482

[31] Archelli S Santillan GI Fonrouge R Céspedes G Burgos L Radman N. Toxocariasis: seroprevalence in abandoned-institutionalized children and infants. Rev Argent Microbiol. (2014) 46:3–6. 10.1016/S0325-7541(14)70040-9 24721267

[32] Santarém VA Pinto GLB de Souza Filho RT Ferreira IB Lescano SAZ Gonzáles WHR Risk factors for toxocariasis during incarceration: the One Health intervention approach. Sci Rep. (2023) 13:19470. 10.1038/s41598-023-45484-7 37945589 PMC10636119

[33] Oliart-Guzmán H Delfino BM Martins AC Mantovani SAS Braña AM Pereira TM Epidemiology and control of child toxocariasis in the western Brazilian Amazon—a population-based study. Am J Trop Med Hyg. (2014) 90:670–81. 10.4269/ajtmh.13-0506 24515946 PMC3973512

[34] BMC. Seroprevalence of *Toxocara Canis* Infection and Associated Risk Factors among Primary Schoolchildren in Rural Southern Thailand|Tropical Medicine and Health|Full Text. Available online at: https://tropmedhealth.biomedcentral.com/articles/10.1186/s41182-020-00211-0 (Accessed June 14, 2025). 10.1186/s41182-020-00211-0 PMC7175560 32336929

[35] Hlushk KT Pavlyshyn HA. Toxocariasis in children with digestive system diseases. Ukr Biochem J. (2022) 94:77–83. 10.15407/ubj94.05.077

[36] Wang S Li H Yao Z Li P Wang D Zhang H *Toxocara* infection: seroprevalence and associated risk factors among primary school children in central China. Parasite. (2020) 27:30. 10.1051/parasite/2020028 32374716 PMC7202827

[37] Kroten A Toczylowski K Kiziewicz B Oldak E Sulik A. Environmental contamination with Toxocara eggs and seroprevalence of toxocariasis in children of northeastern Poland. Parasitol Res. (2016) 115:205–9. 10.1007/s00436-015-4736-0 26385468

[38] Taylan-Ozkan A. Sources and seroprevalence of toxocariasis in Turkey. Adv Parasitol. (2020) 109:465–82. 10.1016/bs.apar.2020.01.021 32381213

[39] Almoslem MM Alshehri TA Althumairi AA Aljassim MT Hassan ME Berekaa MM. Handwashing knowledge, attitudes, and practices among students in eastern province schools, Saudi Arabia. J Environ Public Health. (2021) 2021:6638443. 10.1155/2021/6638443 34567132 PMC8457965

[40] Mendonça LR Figueiredo CA Esquivel R Fiaccone RL Pontes-de-Carvalho L Cooper P Seroprevalence and risk factors for Toxocara infection in children from an urban large setting in Northeast Brazil. Acta Trop. (2013) 128:90–5. 10.1016/j.actatropica.2013.06.018 23845771 PMC7612831

[41] Santarém VA do Couto AC Lescano SZ Roldán WH Delai RR Giuffrida R Serosurvey of anti-*Toxocara canis* antibodies in people experiencing homelessness and shelter workers from São Paulo, Brazil. Parasit Vectors. (2022) 15:373. 10.1186/s13071-022-05499-x 36253837 PMC9574839

[42] Healy S Morgan E Betson M Prada JM. Modelling the risk of food-borne transmission of Toxocara spp. to humans. Epidemiol Infect. (2025) 153:e69. 10.1017/S0950268825000330 40488360 PMC12171901

[43] Kleine A Springer A Strube C. Seasonal variation in the prevalence of Toxocara eggs on children’s playgrounds in the city of Hanover, Germany. Parasit Vectors. (2017) 10:248. 10.1186/s13071-017-2193-6 28526064 PMC5437484

[44] Espinoza YA Huapaya PH Roldán WH Jiménez S Arce Z Lopez E. Clinical and serological evidence of toxocara infection in school children from Morrope District, Lambayeque, Peru. Rev Inst Med Trop S Paulo. (2008) 50:101–5. 10.1590/S0036-46652008000200007 18488089

[45] Otsuka Y Agestika L Harada H Sriwuryandari L Sintawardani N Yamauchi T. Comprehensive assessment of handwashing and faecal contamination among elementary school children in an urban slum of Indonesia. Trop Med Int Health. (2019) 24:954–61. 10.1111/tmi.13279 31192489

[46] Manini MP Marchioro AA Colli CM Nishi L Falavigna-Guilherme AL. Association between contamination of public squares and seropositivity for Toxocara spp. in children. Vet Parasitol. (2012) 188:48–52. 10.1016/j.vetpar.2012.03.011 22480882

[47] Santarém VA Pereira VC Alegre BCP. Contamination of public parks in Presidente Prudente (São Paulo, Brazil) by Toxocara spp. eggs. Rev Bras Parasitol Vet. (2012) 21:323–5. 10.1590/S1984-29612012000300029 23070451

[48] Bradbury RS Hobbs CV. Toxocara seroprevalence in the USA and its impact for individuals and society. Adv Parasitol. (2020) 109:317–39. 10.1016/bs.apar.2020.01.035 32381205

[49] Boleslavsky D Manor U Grossman T Sagi O Ben-Shimol S Schwartz E. Human toxocariasis in Israel: a nationwide serology-based analysis, 2005–2019. Am J Trop Med Hyg. (2022) 106:1265–8. 10.4269/ajtmh.21-0437 35189593 PMC8991344

[50] Merigueti YFFB Santarém VA Ramires LM da Silveira Batista A da Costa Beserra LV Nuci AL Protective and risk factors associated with the presence of Toxocara spp. eggs in dog hair. Vet Parasitol. (2017) 244:39–43. 10.1016/j.vetpar.2017.07.020 28917315

[51] Nijsse R Mughini-Gras L Wagenaar JA Ploeger HW. Recurrent patent infections with *Toxocara canis* in household dogs older than six months: a prospective study. Parasit Vectors. (2016) 9:531. 10.1186/s13071-016-1816-7 27716389 PMC5051026

[52] Bonilla-Aldana DK Morales-Garcia LV Ulloque Badaracco JR Mosquera-Rojas MD Alarcón-Braga EA Hernandez-Bustamante EA Prevalence of Toxocara eggs in Latin American parks: a systematic review and meta-analysis. Infez Med. (2023) 31:329–49. 10.53854/liim-3103-7 37701393 PMC10495062

[53] Giannelli A Schnyder M Wright I Charlier J. Control of companion animal parasites and impact on One Health. One Health. (2024) 18:100679. 10.1016/j.onehlt.2024.100679 39010968 PMC11247265

[54] Rassier GL Borsuk S Pappen F Scaini CJ Gallina T Villela MM Toxocara spp. seroprevalence in sheep from southern Brazil. Parasitol Res. (2013) 112:3181–6. 10.1007/s00436-013-3499-8 23832639

[55] Santarém VA Chesine PAF Lamers BEL Rubinsky-Elefant G Giuffrida R. Anti-Toxocara spp. antibodies in sheep from southeastern Brazil. Vet Parasitol. (2011) 179:283–6. 10.1016/j.vetpar.2011.01.050 21330057

[56] Oliveira Ad Rubinsky-Elefant G Merigueti YFFB Batista AdS Santarém VA. Frequency of anti-Toxocara antibodies in broiler chickens in southern Brazil. Rev Bras Parasitol Vet. (2018) 27:141–5. 10.1590/s1984-296120180025 29846447

[57] von Söhsten AL da Silva AV Rubinsky-Elefant G Guerra LMS de MEM. Anti-Toxocara spp. IgY antibodies in poultry sold in street markets from Feira de Santana, Bahia, Northeastern Brazil. Vet Parasitol Reg Stud Reports. (2017) 8:86–9. 10.1016/j.vprsr.2017.02.006 31014645

[58] Giudice PAF Lescano SAZ Gonzáles WHR Giuffrida R Bandeira FN Kmetiuk LB Serosurvey and associated risk factors of anti-Toxocara spp. antibodies in bovines from slaughterhouses of southeastern Brazil. Parasit Vectors. (2021) 14:250. 10.1186/s13071-021-04755-w 33975623 PMC8111975

[59] Colli CM Rubinsky-Elefant G Paludo ML Falavigna DLM Guilherme EV Mattia S Serological, clinical and epidemiological evaluation of toxocariasis in urban areas of south Brazil. Rev Inst Med Trop Sao Paulo. (2010) 52:69–74. 10.1590/s0036-46652010000200002 20464126

[60] Romasanta A Romero JL Arias M Sánchez-Andrade R López C Suárez JL Diagnosis of parasitic zoonoses by immunoenzymatic assays—analysis of cross-reactivity among the excretory/secretory antigens of *Fasciola hepatica*, *Toxocara canis*, and *Ascaris suum*. Immunol Invest. (2003) 32:131–42. 10.1081/imm-120022974 12916704

[61] Pourgholaminejad A Razipour H Heydarian P Ashrafi K Roushan ZA Sharifdini M. A survey on the seroprevalence of toxocariasis and related risk factors in Eosinophilic children of Northwest Iran. Afr Health Sci. (2022) 22:617–25. 10.4314/ahs.v22i3.66 36910401 PMC9993322

