# Supplementary material for: Toxocariasis in children: seroprevalence after 15 years in a major city in Brazil
Source: Front Pediatr. 2025 Nov 19;13:1663016. doi: 10.3389/fped.2025.1663016 (PMC12672521; doi:10.3389/fped.2025.1663016)

Supplementary Material

Supplementary Figure 1. Educative folder produced by authors containing basic information on toxocariasis and distributed to children/guardians' participants.

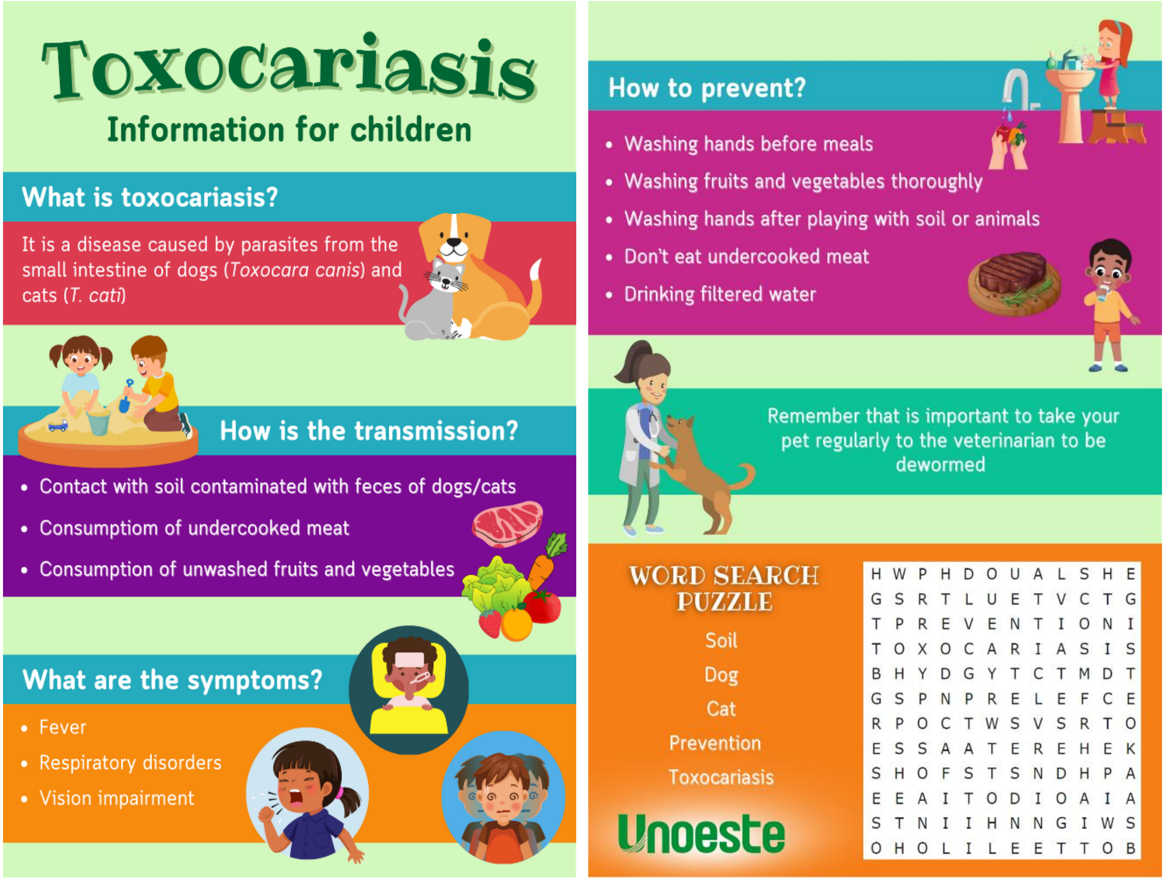

Supplementary Figure 2. Receiver operating characteristic (ROC) curve assessing the accuracy (top; area under curve (AUC): 0.798; 95% CI: 0.712-0.884) of the multivariate logistic regression model for predicting seropositivity for anti-*Toxocara* spp. antibodies in 260 children of southeastern Brazil.

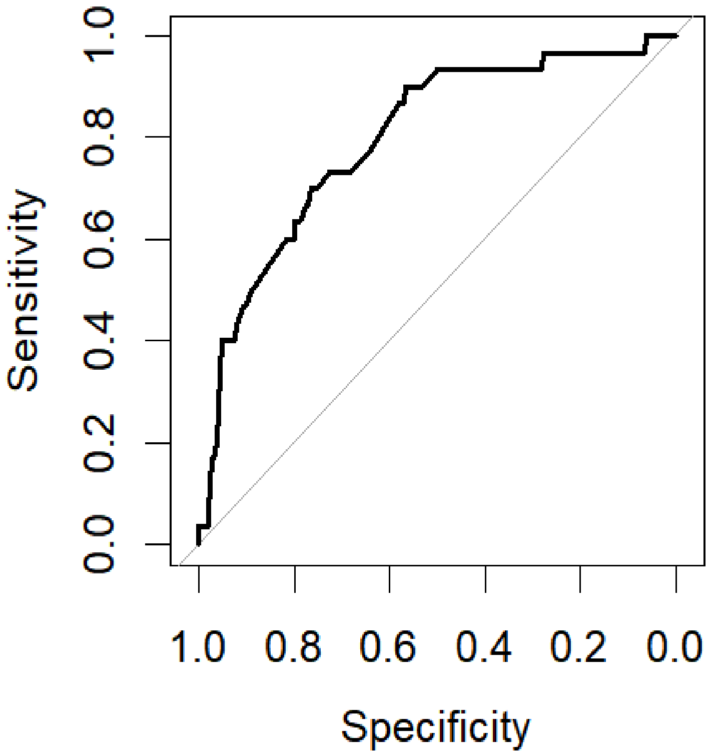

Supplement: Supplementary file 1 [file Datasheet1.pdf]
