# Supplementary material for: Toxocariasis in children: seroprevalence after 15 years in a major city in Brazil
Source: Front Pediatr. 2025 Nov 19;13:1663016. doi: 10.3389/fped.2025.1663016 (PMC12672521; doi:10.3389/fped.2025.1663016)
Supplement: Supplementary file 2 [file Datasheet2.docx]

Supplementary Material

**Supplementary Table 1**. Uni and multivariate analysis for assessing the risk/protective factors associated to *Toxocara* spp. seropositivity in a pediatric population assisted by the Public Health System in southeastern Brazil.

|  | **ELISA test** | | **Statistical Analysis** | | | |
| --- | --- | --- | --- | --- | --- | --- |
|  | **Positive (%)** | **Negative (%)** | **Univariate** | | **Multivariate** | |
| **Analyzed variable** | n=30 (11.5) | n=230 (88.5) | **OR (95% CI)** | ***p*** | **OR (95% CI)** | ***p*** |
| **Gender** |  |  |  | 0.027 |  |  |
| Female | 8 (26.7) | 115 (50.0) | 1.0 [Reference] |  | 1.0 [Reference] |  |
| Male | 22 (73.3) | 115 (50.0) | 2.7 (1.2-6.8) |  | 4.4 (1.8-12.2) | 0.003 |
| **Age** |  |  |  | 0.153 |  |  |
| 1 up to 5 | 8 (26.7) | 97 (42.2) | 1.0 [Reference] |  |  |  |
| 6 up to 11 | 22 (73.3) | 133 (57.8) | 2.0 (0.9-5.0) |  |  |  |
| **Household location** |  |  |  | 0.096 |  |  |
| Rural | 6 (20.0) | 20 (8.70) | 1.0 [Reference] |  |  |  |
| Urabn | 24 (80.0) | 210 (91.3) | 0.4 (0.1-1.1) |  |  |  |
| **Maternal education level** |  |  |  | 0.170 |  |  |
| Elementary | 8 (26.7) | 34 (14.8) | 1.0 [Reference] |  | 1.0 [Reference] |  |
| High School | 16 53.3) | 121 (52.8) | 0.6 (0.2-1.5) |  | 0.5 (0.2-1.3) | 0.137 |
| Graduate | 6 (20.0) | 74 (32.3) | 0.4 (0.1-1.1) |  | 0.2 (0.1-0.7) | 0.016 |
| **Monthly income** |  |  |  | 0.267 |  |  |
| ≤1 minimum wage | 16 (57.1) | 96 (44.0) | 1.0 [Reference] |  |  |  |
| ≥1 minimum wage | 12 (42.9) | 122 (56.0) | 1.7 (0.8-3.8) |  |  |  |
| **Drinkable water supply** |  |  |  | 0.004 |  |  |
| No | 8 (26.7) | 18 (7.83) | 1.0 [Reference] |  | 1.0 [Reference] |  |
| Yes | 22 (73.3) | 212 (92.2) | 0.2 (0.1-0.6) |  | 0.3 (0.1-0.9) | 0.033 |
| **Unpaved yard at house** |  |  |  | 0.346 |  |  |
| No | 13 (43.3) | 125 (54.3) | 1.0 [Reference] |  |  |  |
| Yes | 17 (56.7) | 105 (45.7) | 1.6 (0.7-3.4) |  |  |  |
| **Playing on playgrounds** |  |  |  | 0.893 |  |  |
| No | 9 (30.0) | 62 (27.0) | 1.0 [Reference] |  |  |  |
| Yes | 21 (70.0) | 168 (73.0) | 0.9 (0.4-2.1) |  |  |  |
| **Having dog** |  |  |  | 0.158 |  |  |
| No | 6 (20.0) | 80 (34.8) | 1.0 [Reference] |  | 1.0 [Reference] |  |
| Yes | 24 (80.0) | 150 (65.2) | 2.1 (0.9-5.9) |  | 2.1 (0.8-6.5) | 0.162 |
| **Contact with dog** |  |  |  | 0.182 |  |  |
| No | 2 (6.67) | 42 (18.3) | 1.0 [Reference] |  |  |  |
| Yes | 28 (93.3) | 188 (81.7) | 2.9 (0.8-20.1) |  |  |  |
| **Having cat** |  |  |  | 0.654 |  |  |
| No | 21 (70.0) | 174 (75.7) | 1.0 [Reference] |  |  |  |
| Yes | 9 (30.0) | 56 (24.3) | 1.3 (0.6-3.0) |  |  |  |
| **Contact with cat** |  |  |  | 0.138 |  |  |
| No | 14 (46.7) | 144 (62.6) | 1.0 [Reference] |  |  |  |
| Yes | 16 (53.3) | 86 (37.4) | 1.9 (0.9-4.2) |  |  |  |
| **Onychophagy** |  |  |  | 0.27 |  |  |
| No | 14 (46.7) | 136 (59.1) | 1.0 [Reference] |  |  |  |
| Yes | 16 (53.3) | 94 (40.9) | 1.7 (0.8-3.6) |  |  |  |
| **Pica** |  |  |  | 0.764 |  |  |
| No | 12 (40.0) | 103 (44.8) | 1.0 [Reference] |  |  |  |
| Yes | 18 (60.0) | 127 (55.2) | 1.2 (0.6-2.7) |  |  |  |
| **Ingestion of raw/undercooked meat** |  |  |  | 0.268 |  |  |
| No | 22 (84.6) | 189 (91.7) | 1.0 [Reference] |  |  |  |
| Yes | 4 (15.4) | 17 (8.25) | 2.1 (0.5-6.3) |  |  |  |
| **Washing hands before meals** |  |  |  | 0.016 |  |  |
| No | 10 (33.3) | 33 (14.4) | 1.0 [Reference] |  | 1.0 [Reference] |  |
| Yes | 20 (66.7) | 196 (85.6) | 0.3 (0.2-0.8) |  | 0.3 (0.1-0.7) | 0.008 |
